# Successful editing and maintenance of lactogenic gene expression in primary bovine mammary epithelial cells

**DOI:** 10.1007/s11626-023-00762-6

**Published:** 2023-06-06

**Authors:** Janelle Moody, Emily Mears, Alexander J. Trevarton, Marita Broadhurst, Adrian Molenaar, Thaize Chometon, Thomas Lopdell, Matthew Littlejohn, Russell Snell

**Affiliations:** 1 Applied Translational Genetics Group, School of Biological Sciences, University of Auckland, Auckland, New Zealand; 2 Ruakura, AgResearch Ltd, PB 3123 Hamilton, New Zealand; 3 Grasslands, AgResearch Ltd, Palmerston North, New Zealand; 4 Faculty of Sciences, Auckland Cytometry, The University of Auckland, Auckland, New Zealand; 5 Livestock Improvement Corporation, Hamilton, New Zealand

**Keywords:** Primary bovine mammary epithelial cells, CRISPR-Cas9, Matrigel, DGAT1, FACS

## Abstract

In vitro investigation of bovine lactation processes is limited by a lack of physiologically representative cell models. This deficiency is most evident through the minimal or absent expression of lactation-specific genes in cultured bovine mammary tissues. Primary bovine mammary epithelial cells (pbMECs) extracted from lactating mammary tissue and grown in culture initially express milk protein transcripts at relatively representative levels. However, expression drops dramatically after only three or four passages, which greatly reduces the utility of primary cells to model and further examine lactogenesis. To investigate the effects of alternate alleles in pbMECs including effects on transcription, we have developed methods to deliver CRISPR-Cas9 gene editing reagents to primary mammary cells, resulting in very high editing efficiencies. We have also found that culturing the cells on an imitation basement membrane composed of Matrigel, results in the restoration of a more representative lactogenic gene expression profile and the cells forming three-dimensional structures in vitro. Here, we present data from four pbMEC lines recovered from pregnant cows and detail the expression profile of five key milk synthesis genes in these MECs grown on Matrigel. Additionally, we describe an optimised method for preferentially selecting CRISPR-Cas9-edited cells conferring a knock-out of DGAT1, using fluorescence-activated cell sorting (FACS). The combination of these techniques facilitates the use of pbMECs as a model to investigate the effects of gene introgressions and genetic variation in lactating mammary tissue.

**Supplementary Information:**

The online version contains supplementary material available at 10.1007/s11626-023-00762-6.

## Introduction

Physiologically relevant modelling of biological processes in cultured cells, particularly those isolated from terminally differentiated tissues, is challenging. Cells obtained from biopsies lose their specific gene expression profiles after a small number of divisions in vitro (Jedrzejczak and Szatkowska 2014). Changes in cell type representation can further complicate the culture of somatic cells for functional modelling. For example, some cell types, such as fibroblasts, can become the dominant cell population because of their relatively rapid division in a mixed cell culture (Pal and Grover 1983). The immortalisation of cells isolated from somatic tissue facilitates an infinite window in which cells can be cultured but often leads to compromised characteristics not representative of the primary tissue (Zhou *et al*. 2008). A potential solution to this problem is to apply a new methodology to enable the extended use of primary cells while they remain in their pre-immortalised state.

There has been considerable effort to uncover genetic and biochemical influences on bovine lactational traits using in vitro cell models of mammary tissue (Kumura *et al*. 2001; Sakamoto *et al*. 2005; Hernandez *et al*. 2008). The most frequently used models are immortalised cell lines, such as MAC-T (Huynh *et al*. 1991) or BME-UV (Zavizion *et al*. 1996), which maintain the ability to proliferate. However, after multiple generations in culture, these cells lack many important, physiologically relevant lactogenic characteristics such as milk protein expression. In contrast, primary bovine lactating mammary epithelial cells (pbMECs) in culture initially have high milk protein gene expression levels. Unfortunately, this phenotype changes significantly over a few passages, where expression of milk protein genes often reduces and becomes undetectable (Jedrzejczak and Szatkowska 2014). During the culturing of pbMECs, lactogenic hormones such as dexamethasone and prolactin are added to culture media to induce or help maintain some level of the lactogenic phenotype (Kumura *et al*. 2001; Sakamoto *et al*. 2005; Zhao *et al*. 2010).

Furthermore, the growth of pbMECs on an artificial basement membrane and the formation of three-dimensional (3D) structures has been shown to support the differentiation of pbMECs into ductal and luminal epithelial cells (Rose *et al*. 2002; Boutinaud *et al*. 2015). This formation of 3D structures is associated with a more sustained profile of alpha, beta, and kappa casein gene expression compared to cells grown in 2D (Hillreiner *et al*. 2017). Matrigel is a complex solution containing many extracellular matrix proteins secreted from mouse sarcoma cells (Kibbey 1994). When attached to plastic culture dishes, it mimics the basement membrane and facilitates the attachment of cultured cells. The structural support of the Matrigel is thought to trigger differentiation and return epithelial cells to a more lactogenic state compared to monolayer growth on plastic (Mroue and Bissell 2013).

The advent of efficient Clustered Regularly Interspaced Short Palindromic Repeat – Cas9 (CRISPR-Cas9) targeted editing has enabled the dissection of phenotypic consequences due to genetic changes at specific sites (Liu *et al*. 2019b). The CRISPR-Cas9 system is a multifunctional tool that is now frequently used to edit genomes, allowing the researcher to assess the effects of the induced genetic changes on gene function or expression in many model systems (Ebrahimi and Hashemi 2020). To date, there have only been a few reports describing the use of gene editing to investigate the effects of genetic variants on lactogenic gene expression in lactating mammary epithelial cells (Tian *et al*. 2018; Huang *et al*. 2020; Edick *et al*. 2021). A major limitation of this research is that the number of cell divisions required to complete experiments is often outside the window of physiologically representative gene expression in pbMECs. A number of factors influence the editing efficiency of the CRISPR-Cas9 complex; the activity of the guide RNA, the number of complexes a cell is transfected by, and the availability of the genomic region being edited. This intrinsic variation is particularly concerning when a heterogeneous population of cells rather than a clonal population is to be analysed (Fu *et al*. 2021).

Here, we present a combined protocol, which describes the culture and characterisation of pbMECs and their transfection and editing, using the critical gene for lactogenesis, *DGAT1,* as an example of this methodology. We demonstrate how fluorescence-activated cell sorting (FACS) can greatly improve the frequency of edited cells in the final cell population. We also show that growth of the sorted CRISPR-Cas9-edited pbMCEs on Matrigel improves the maintenance of lactogenic gene expression. The combination of these methodologies opens the window for the testing of gene variant function in primary cell lines.

## Materials and Methods

### Establishing an epithelial cell line

pbMECs were derived from parenchymal mammary tissue obtained after slaughter, from four cows at 6 to 7 mo of their first pregnancy. The animals were obtained from the Ruakura Dairy Farm and were slaughtered in accordance with MPI regulations at the AgResearch abattoir on the Ruakura Farm, under the AgResearch ethics approval number 3925. The Ruakura Animal Ethics Committee approved the study. These methods are in accordance with the ARRIVE guidelines. The tissue was washed in Hanks’ Balanced Salt Solution (10 ×) (HBSS) and then digested using Digest Medium (Table 1.) for 2 h at 37 °C. The tissue was then minced using sterile curved scissors and scalpel blades and put back into Digestion Medium for 3 h to remove all other connective tissue. Cells were filtered through a 150 μm mesh filter and washed three times in Wash Medium (Table 1.) at 100 × g for 10 min, after which cells were resuspended in 5 mL of HBSS. Finally, cells were fractionated by centrifugation at 800 g for 20 min using a Percoll gradient with six fractions. Epithelial cells from the 1.03–1.05 (g/mL) fraction were removed and washed in HBSS (10 ×). These cell harvest and purification steps were performed in 2001, and the resultant cells were subsequently frozen in 20% DMSO and stored in liquid nitrogen. Revival of cells was conducted by suspension of the vial in a 37 °C water bath until no ice remained. Thawed cells were added to proliferation media (10% foetal bovine serum (FBS), 1% penicillin/streptomycin, 5 µg/mL insulin, 1 µg/mL progesterone, Dulbecco’s Modified Eagle Medium (DMEM)) and pelleted at 500 × g for 5 min. Cells were then added to differentiation media (DM) containing 10% FBS, 1% penicillin/streptomycin, 5 µg/mL insulin, 10 µg/mL dexamethasone, and 5 µg/mL prolactin in DMEM. DM is used to stimulate the cells to differentiate into a more lactogenic state. The lines used for subsequent experiments are referred to in the manuscript as the Pink, Orange, Green, and Brown lines.

**Table 1.** Reagents used in the cell culture media for the extraction of pbMECs

	Amount (mL)	Final concentration		Amount	Final concentration
Digest base			Digest medium		
Hanks (10 ×)	50	1x	Digest base	499 mL	
MEAA	10	-	Insulin (1 mg/mL)	2.5 mL	5 μg/mL
Glucose (100 mM)	27.5	5.5 mM	Cortisol (5 mg/mL)	100 μL	1 μg/mL
BSA (10% w/v)	200	4%	Collagenase	600 mg	1.2 mg/mL
Ca/Mg (4.65 mg/mL)	0.5	4.65 μg/mL	Hyaluronidase	250 mg	0.5 mg/mL
Hepes (1 M)	2.5	5 mM			
Glutamine (200 mM)	5	2 mM			
Fungizone (25 μg/mL)	5	0.25 μg/mL			
Kanamycin (10 mg/mL)	5	100 μg/mL			
Water	243.5				
Total	499				
Wash base			Wash medium		
Hanks (10 ×)	50	1 ×	Wash base	499 mL	
MEAA	10	-	Insulin (5 mg/mL)	0.5 mL	5 μg/mL
Glucose (100 mM)	27.5	5.5 mM	Cortisol (5 mg/mL)	100 μL	1 μg/mL
Ca/Mg (4.65 mg/mL)	0.5	4.65 μg/mL	DNAse I	10 mg	20 μg/mL
Hepes (1 M)	2.5	5 mM	Trypsin Inhibitor	50 mg	100 μg/mL
Glutamine (200 mM)	5	2 mM			
Fungizone (25 μg/mL)	5	0.25 μg/mL			
Antibiotics (22 mg/mL)	5	0.22 mg/mL			
Water	393.5				
Total	499				

### Immunocytochemistry

All cell lines were seeded at 300,000 cells per well, and grown to 95% confluency for five passages in a 6-well plate before being fixed in the well with ice cold, 100% methanol. Cells were incubated with 1% BSA, 22.52 mg/mL glycine in PBST (PBS + 0.1% Tween 20) to block non-specific antibody binding before being incubated for 1 h at room temperature in a 1:1000 dilution of the Anti-Cytokeratin 8 primary antibody [C-43] (FITC) (ab176533). Following a PBS wash, the cells were incubated with the secondary antibody, Alexa Fluor 594 goat anti-mouse IgG (H + L) A11032, at a final concentration of 2 μg/mL in 1% BSA for 1 h at room temperature in the dark. Cells were then counterstained with DAPI at a final concentration of 1 μg/mL before visualisation using a Nikon Ti-E inverted microscope and TxRed (542–644 nm) and DAPI (352–477 nm) fluorescence filter cube.

### qPCR of key milk-related gene expression

The expression of five key milk genes was assessed over three consecutive passages (P1-P3) following the thawing of stored pbMEC aliquots. Cells were grown until 90% confluent in one well of a 12-well plate before digestion with Trypsin–EDTA (0.25%) (Gibco - Carlsbad, CA, USA). RNA was extracted from half of the harvested cells from each well using an RNeasy kit (Qiagen - Hilden, Germany) while re-seeding the remaining half into one well of a new 12-well plate. cDNA was generated from RNA using SuperScript™ III First-Strand Synthesis SuperMix (Invitrogen), and subsequently, gDNA contamination was removed using the DNA-free™ Kit (Life Technologies). qPCR assays for relative RNA quantification were undertaken using the Roche Universal Probe Library system on a LightCycler 480 machine for five target genes *GPAT4* (formerly *AGPAT6*)*, CSN2, DGAT1, PAEP* (formerly *LGB*), and *MGST1* and two reference genes *EIF3K* and *RPS15A* (Walker *et al*. 2009). Details of the qPCR primers, probes, and expected amplicon sizes can be found in Table 2.. Delta-Delta-Ct analysis was performed on the raw Ct results using the average results from passage one in each line as the control. A one-tail two-sample equal variance *t*-test was used to calculate the *p*-values for the difference in expression between the three passages in each cell line. These data are present in the supplementary materials (Supplementary Table 1). All raw data and calculations can be found at https://doi.org/10.7910/DVN/YATECZ.

**Table 2.** Primer sequences (5′ to 3′), Roche Universal probes, and expected amplicon sizes for qPCR of key lactogenic genes. *bp*, base pairs

Gene	Primer sequence	Roche probe #	Amplicon size (bp)
*GPAT4*	FWD: GCTCCGAAGTGAAGGATCG	49	65
	REV: GCTTTTATCCTGCACATGCTC		
*CSN2*	FWD: GGGCATTCACTTTGAAATCCT	92	60
	REV: ATATGCCCATTCAGGCCTTT		
*DGAT1*	FWD: TCTGTGCCTGGTCATTG	140	102
	REV: CGCTTCTCCACCTGGAAC		
*PAEP*	FWD: CCCCCTGAGAGTGTATGTGG	165	92
	REV: GAGCACACTCACCGTTCTCC		
*MGST1*	FWD: GCTTCGGCAAAGGAGAAA	142	96
	REV: CGATGTTTTCAAGGTCATTCAAG		
*EIF3K*	FWD: AAGTTGCTCAAGGGGATCG	1	77
	REV: TTGGCCTGTGTCTCCACATA		
*RPS15A*	FWD: TCAGCCCTAGATTTGATGTGC	32	104
	REV: GCCAGCTGAGGTTGTCAGTA		

### Estimating the transfection and editing efficiency of pbMECs

To assess their transfection efficiency, pbMECs at passage 6 were removed from the plastic well using Trypsin–EDTA (0.25%) and seeded in differentiation media into one well of a 24-well plate at 100,000 cells per well in 443 μL of media. Immediately after plating, while the cells were still suspended in the media, they were reverse-transfected, utilising lipofection to deliver the tracrRNA. The reactions consisted of 0.2 μM of ATTO-labelled tracrRNA (IDT, Cat:1,075,927) 2.5 μL of Cas9 reagent, 1.5 μL of CRISPRMAX (Invitrogen), and 50 μL of Opti-MEM. Following the addition of the transfection reagents, the cells were grown for 24 h before visualising the ATTO-labelled tracrRNA using the Nikon Ti-E inverted microscope.

To investigate gene editing of pbMECs using the CRISPR/Cas9 system, the CRISPRMAX reagents were used to reverse transfect a ribonucleoprotein (RNP) complex comprised of the *S. pyogenes* Cas9 nuclease complexed with one of two different guide RNAs. These guides were designed to cut in two locations and potentially delete a region of *DGAT1* exon 1, thereby knocking out expression. The guides were designed using the web-based software CRISPOR, which finds potential guide RNA sequences and ranks them based on their predicted activity and potential off-target effects (Concordet and Haeussler 2018). The crRNA sequences were as follows: guide 1—/AltR1/rGrCrUrArCrGrArCrUrUrGrGrCrCrGrCrGrGrCrGrGrUrUrUrUrArGrArGrCrUrArUrGrC rU/AlTR2/, guide 2—/AltR1/rArArGrGrArCrGrGrArGrArCrGrUrArGrArCrGrUrGrUrUrUrUrArGrArG rCrUrArUrGrCrU/AltR2/, where AltR1 and AltR2 are end-blocking modifiers. The crRNAs were synthesised by IDT. Again, the Invitrogen CRISPRMAX protocol was used to make up the appropriate transfection reactions for a 24-well plate (see above). The RNP complex was assembled at a molar ratio of 1:1.6 guide RNA:Cas9 Protein (IDT, Cat:1,081,058) with a final concentration of 200 nM for each reaction, in a final volume of 500 μL. Cells were seeded in a 24-well plate at 100,000 cells per well before reverse transfection with the RNP complex. After the addition of the editing reagents, the cells were grown for 48 h before they were removed by digestion with 2 mL of Trypsin and DNA extracted using a DNeasy column kit (Qiagen). PCR was undertaken with primers (P1 & P2) flanking the Cas9-mediated cleavage sites (Primer P1: 5′ CAGTTGGCCAAGGGTCCG 3′ and Primer P2 5′ AGGGGTCAAAGGTTAGGGGT 3′). The PCR cycling conditions were as follows: initial denaturation at 95 °C for 3 min, denaturation at 95 °C for 15 s, annealing at 56 °C for 15 s and extension at 72 °C for 15 s for 35 cycles, and a final extension at 72 °C for 1 min. The wild-type product was expected to be 322 bp in length, and the double cut product was expected to be 138 bp.

### Culturing pbMECs in 3D

The imitation basement membrane used to coat the plates, Matrigel (Corning, NY), was prepared by 2 × dilution with serum-free DMEM media (Xu and Buchsbaum 2012). Twenty-four-well plate wells were coated with 200 μL of diluted Matrigel by spreading onto the bottom of the wells and incubating at 37 °C for 30 min to solidify. The initial qPCR experiment revealed that the Green pbMEC line had consistent expression of all target genes (Fig. 4). Therefore, the Green pbMEC line was selected for 3D culturing experiments.

**Figure 1.**
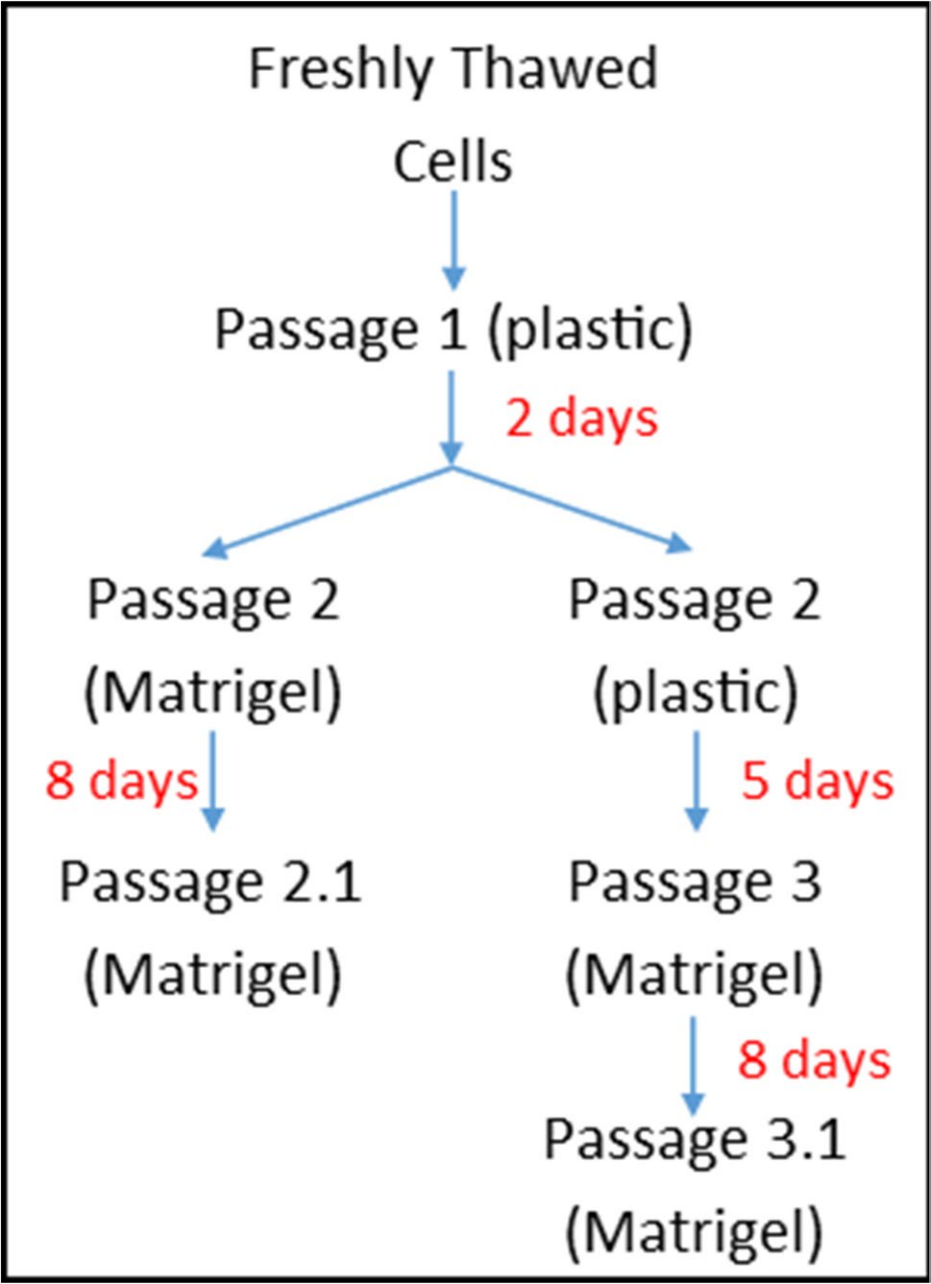
**pbMEC passaging** **workflow. **Flow chart showing the designation of the passages and culturing conditions undertaken with the Green pbMEC cell line comparing plastic and Matrigel growth substrates. RNA was extracted from a proportion of the cells grown at each passage for subsequent analysis.

Cells stored in liquid nitrogen were thawed and plated on plastic in differentiation media to recover for 2 d (Fig. 1). After 2 d of growth on plastic, 2 × 10^5^ of these Passage 1 cells were plated onto Matrigel, grown for 8 d, removed from the Matrigel using 600 μL of cell recovery solution (Corning) to keep the 3D structures intact, and re-seeded onto Matrigel. These cells were grown for a further 8 d before extraction from the wells using the cell recovery solution. In parallel, 3 × 10^5^ passage 1 cells were re-seeded back onto a plastic 6-well plate where they were grown for 5 d before following the protocol as above, with two more passages completed on Matrigel. Each passage from plastic to plastic, or plastic to Matrigel is denoted as P1, P2, or P3. Passaging from Matrigel to Matrigel is denoted as P2.1 and P3.1 (see Fig. 1 for details). A one-tail two-sample equal variance *t*-test was used to calculate the *p*-values for the difference in expression seen between passage 2 cells grown on plastic or Matrigel in Fig. 7.

### *DGAT1* knock-out via CRISPR/Cas9 editing

**Figure 2.**
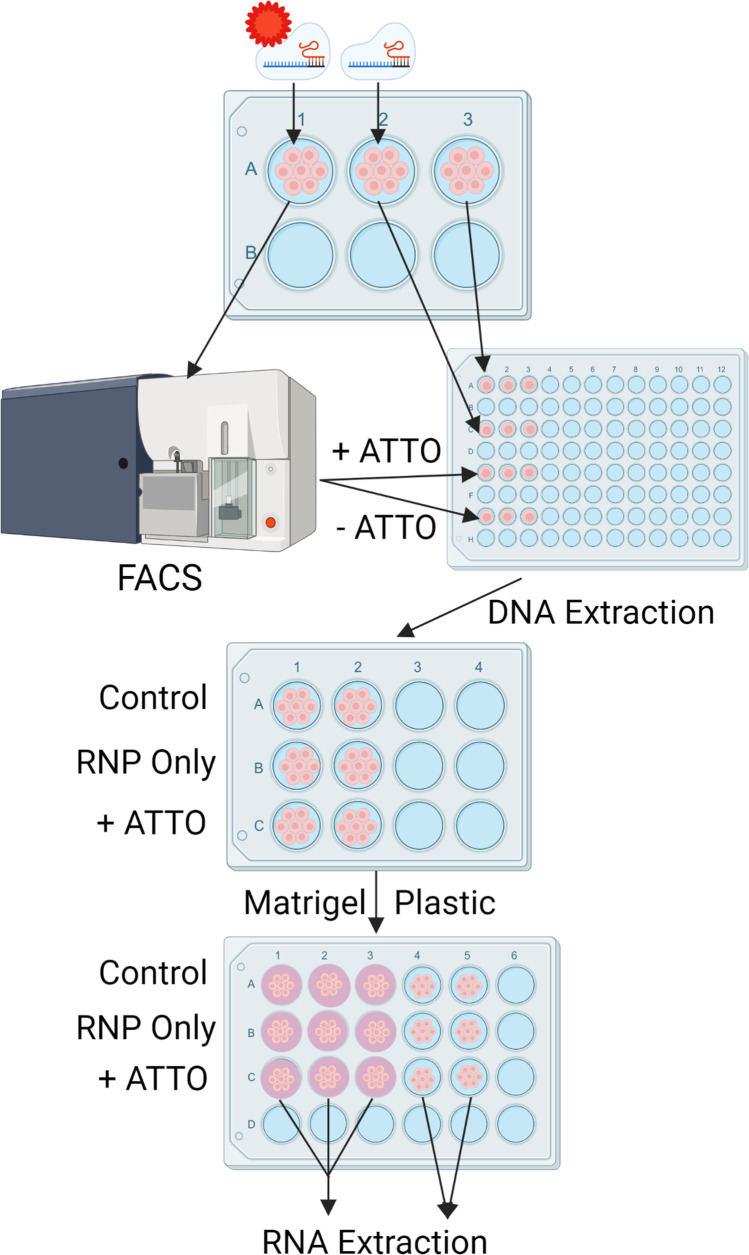
**Workflow for editing, FACS sorting and qPCR of pbMECs.** Cells are transfected with either the RNP complex plus ATTO-labelled tracrRNA or the RNP complex alone in a 6-well plate. The first well was sorted using FACS into ATTO positive and ATTO negative cell populations. All groups of cells were transferred into a 96-well plate and grown to 95% confluency before being transplanted to a 12-well plate for outgrowth. At this point, DNA was extracted from all cell populations. Once confluent in the 12-well plate, the cells were pooled and plated onto a 24-well plate in three Matrigel-coated wells and two plastic wells. After 2 wk for Matrigel wells and 5 d for plastic wells, cells of each population, in each condition (Matrigel and plastic) were pooled, and RNA was extracted for qPCR.

An initial qPCR experiment measuring the relative expression of milk production genes in the 4 primary lines revealed that the Brown pbMEC line had the highest expression of CSN2 and LGB (relative expression was 4.84 and 2.9, respectively) after the first passage, suggesting the retention of a physiologically relevant phenotype. An aliquot of these cells was thawed and passaged once and then 3 wells of a 6-well plate were seeded into differentiation media, with 1 × 10^6^ cells in each well (Fig. 2). Wells A1 and A2 of the 6-well plate were reverse transfected by CRISPRMAX (12.5 μL of Cas9 reagent, 7.5 μL of CRISPRMAX (Invitrogen), and 250 μL of Opti-MEM). The transfection introduced an RNP complex containing *DGAT1* knock-out dual guide RNAs, at a final concentration of 200 nM for both guides combined, in a total volume of 2 mL. The liposomes in the A1 also contained ATTO-labelled tracrRNA at a final concentration of 200 nM, which was co-transfected in addition to the RNP complex. The second well (A2) was only transfected with the RNP complex, and the third well (A3) of cells was a non-transfected control. After 24 h in culture, the cells were detached using Trypsin–EDTA (0.25%) and 1 × 10^4^ cells from wells A2 and A3 were each plated in three wells of a 96-well plate. The ATTO-transfected cells (well A1) were FACS sorted on a BD FACS Aria II SORP (BD) after using DAPI counterstaining for live cell selection, and using 550 nm ATTO positive signal to sort them into positive and negative pools. These cells were plated into three 96-well plate wells at 1 × 10^4^ cells per well and grown to confluency. Once confluent, the cells from each treatment (3 wells) were pooled for each condition, transferred into a 12-well plate, and grown to confluency. Once 95% confluent, cells from the 12-well plate (edited and sorted ATTO positive cells, edited unsorted cells and control cells) were detached, and 1 × 10^5^ cells per well were seeded into a 24-well plate, into wells that were either plain plastic (two wells) or were coated with Matrigel (three wells). The remaining cells were used for DNA extraction using a DNeasy column kit (Qiagen). PCR was undertaken with different primers (P1_Long & P2_Long) flanking the Cas9-mediated cleavage sites (Primer P1_Long: 5′ CAGCGGACTACAAAGGTATG 3′ and Primer P2_Long 5′ CAACCTCCCGCTAAGTTTC 3′). The wild-type product was expected to be 618 bp in length, and the double cut product was expected to be 434 bp. The KAPA Taq PCR Kit (Merck, Cat: KK1008) was used to complete PCR. These amplicons were sequenced via MinION sequencing (Oxford Nanopore Technologies) which was performed per the MinION 1D Native barcoding genomic DNA protocol, using Ligation sequencing kit (SQK-LSK109) and Barcoding expansion kit (EXP-NBD103).

The cells plated on plastic were cultured for 5 d, and the cells cultured on Matrigel were grown for 2 wk before harvesting using 2 mL of trypsin for the plastic wells and 600 μL of Corning Cell Recovery Solution for the Matrigel wells. RNA was extracted using a Qiagen RNeasy Kit. Cells from the two or three wells for each condition were pooled to ensure sufficient RNA was extracted; that is, wells were not treated as biological replicates. qPCR of the target *DGAT1* and the two reference genes *EIF3K* and *RPS15A* was then conducted on all samples to determine the relative expression of *DGAT* in the ATTO positive, RNP only and wild-type cell populations. qPCR was also completed in a preliminary experiment with these methods, and relative expression of *GPAT4* and *MGST1* genes was assessed. Delta-Delta-Ct analysis was performed on the raw Ct results using the 125 × dilution from the standard curve as the control.

### Analysis of potential off target editing sites

The cutting frequency determination (CFD) score of the CRISPOR software was used to identify the top four ranked off-target regions for each guide (Liu *et al.* 2019a). PCR primer sets designed for these off-target regions can be found in Supplementary Table 2. The iProof™ High-Fidelity PCR Kit (Bio-Rad, Cat: 1,725,330) was used to complete PCR on the ATTO plus RNP (well A1) DNA sample from the DGAT1 knock-out via CRISPR/Cas9 editing experiment. The PCR cycling conditions were as follows: initial denaturation at 98 °C for 30 s, denaturation at 98 °C for 5 s, annealing at 59 °C for 10 s and extension at 72 °C for 15 s for 32 cycles, and a final extension at 72 °C for 5 min. The amplicons were cleaned using a PCR clean up kit (Machery-Nagel, Cat: MN740609.250) and sequenced via Sanger sequencing.

### Equipment and settings

**Figure 3.**
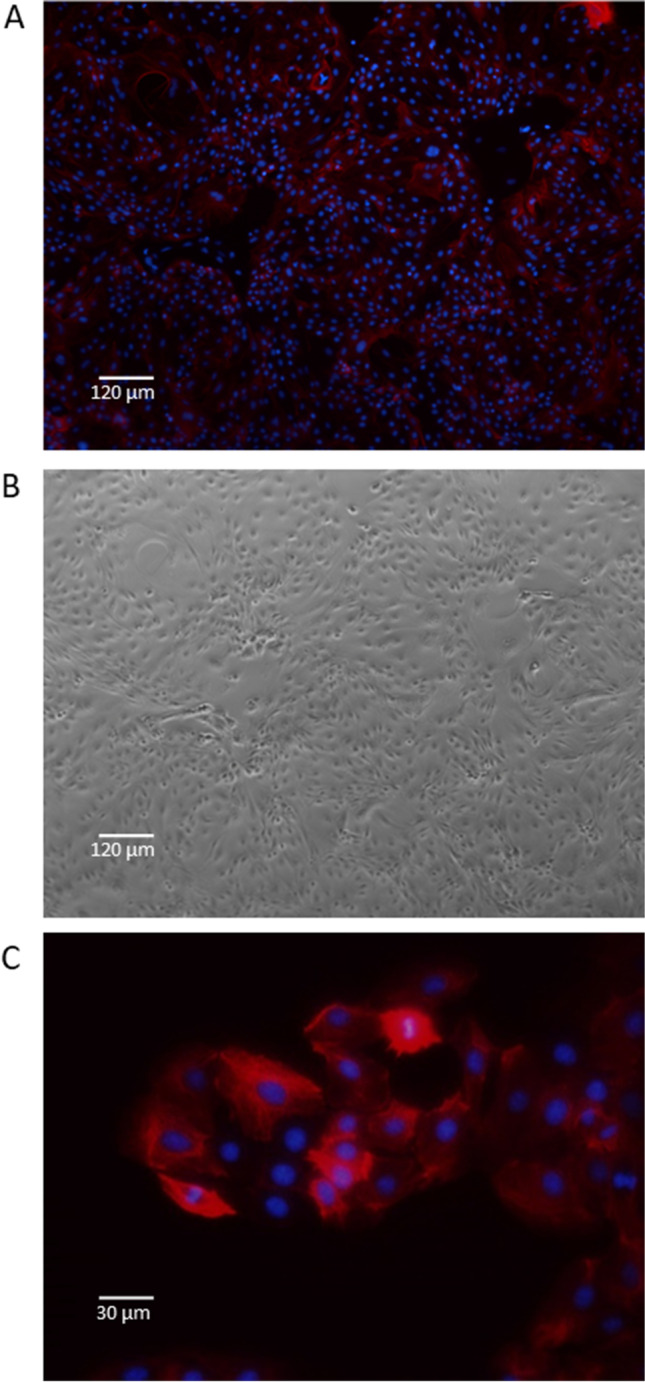
**Visualisation of fixed and stained Brown pbMEC line. **Cytokeratin-8 and nuclei staining of fixed pbMECs demonstrates typical epithelial cell morphology. (*A*) pbMECs incubated with primary cytokeratin 8 antibody (ab176533) and Alexa Fluor 594 goat anti-mouse secondary (A11032), counterstained with DAPI at 10 × magnification. (*B*) Bright-field image of the same frame as in A. (*C*) pbMECs incubated with primary cytokeratin 8 antibody (ab176533) and Alexa Fluor 594 goat anti-mouse secondary (A11032), counterstained with DAPI at 40 × magnification.

All agarose gels were imaged on a Bio-Rad Gel Doc XR + using Image Lab™ software (version 5.2.1). The ‘GelRed’ application was used, and the exposure time was optimised for faint bands. The microscopy images were taken on a Nikon Ti-E inverted microscope using NIS-Elements software (Nikon Instruments Inc). TxRed (542–644 nm) and DAPI (352–477 nm) fluorescence filter cubes were used to visualise the Alexa Fluor 594 goat anti-mouse IgG (H + L) A11032 and DAPI stain. Photoshop was used to enhance the brightness and contrast of the red and blue fluorescence in Fig. 3*A* and *C*. This was applied evenly across the entire image.

## Results

### Epithelial characterisation

**Figure 4.**
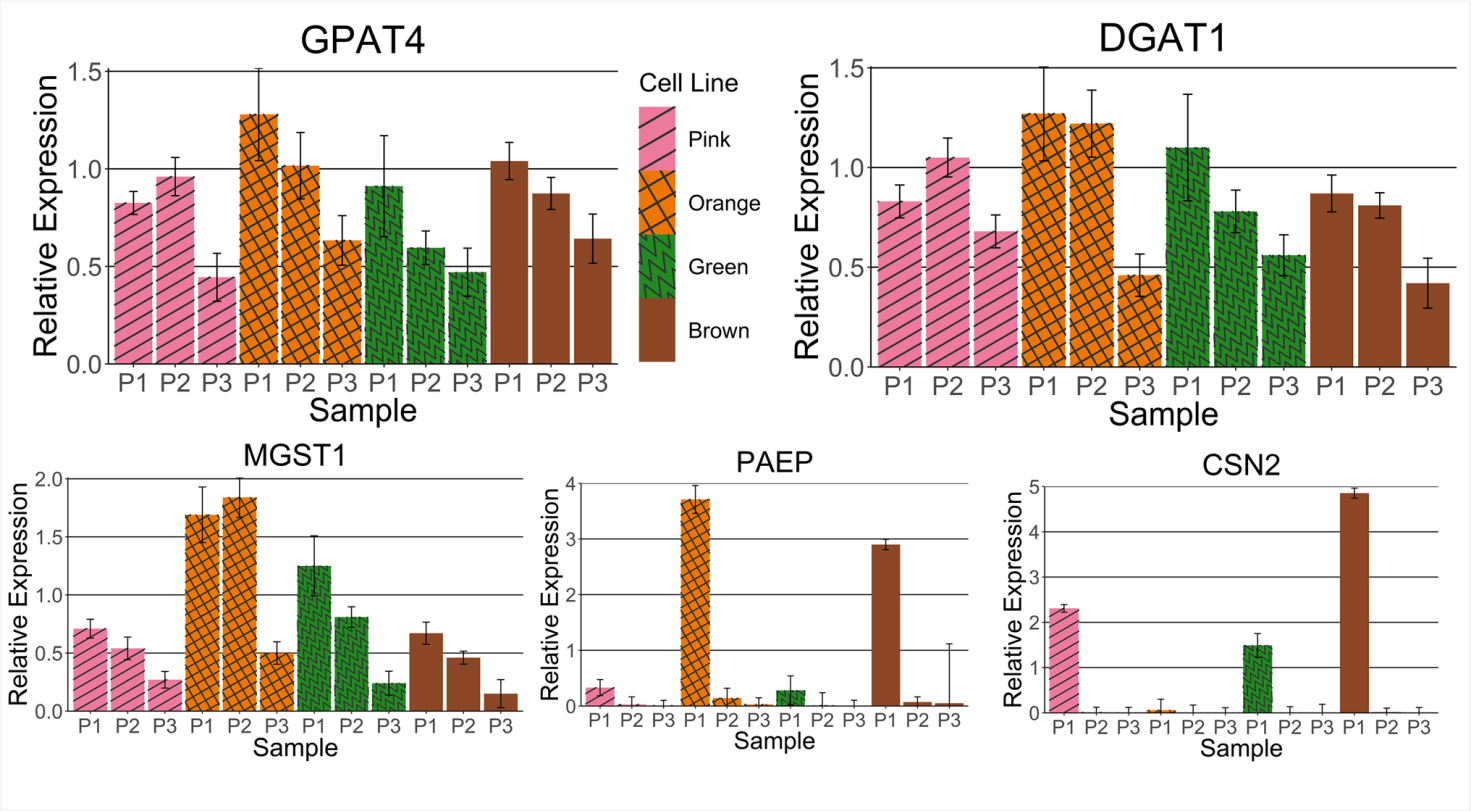
**Relative expression of lactogenic** **genes decreases through passages of four different pbMEC cell lines.** Relative expression of five lactogenic genes (*GPAT4, DGAT1, MGST1, CSN2*, and *PAEP*) in four different pbMEC lines grown on plastic across three passages labelled P1-P3. The average Ct value for passage 1 for all the lines was used as the relative expression level control. The expression results of four cell lines (*pink*, *orange*, *green*, and *brown*) are indicated by their respective colours. *Error bars* show one standard deviation.

After thawing the cryopreserved pbMECs, the cells from all investigated lines were aggregated in the culture media. It took 2 d for these clumps to settle on the bottom of the well and for the epithelial cells to migrate out and form a monolayer. Once confluent, the cells were passaged using trypsin to detach them from the well surface; this also served to disaggregate the clusters further, resulting in a more dispersed pool of cells in the following passage. The pbMECs were grown to passage 5 in one well of a 6-well plate, by which time they grew exclusively as a monolayer and were fixed with methanol for immunocytochemistry (ICC). Cells were stained with a mouse anti-cytokeratin 8 primary antibody and a goat anti-mouse IgG secondary antibody conjugated to a red-fluorescent dye Alexa Fluor 594. Cytokeratin 8 is a cytoskeletal protein shown to identify mammary epithelial cells (Boutinaud *et al*. 2015). The majority of cells exhibited red staining in the cytoplasm, indicating that the cells were mainly mammary epithelial cells as expected (Fig. 3*A*, *B*, and *C*). To assess the relative gene expression levels of key lactation genes across time in the pbMECs, qPCR was conducted on five target genes. These were triglyceride synthesis genes *GPAT4* and *DGAT1*; *CSN2* and *PAEP*, which are components of milk casein and whey protein, respectively; and *MGST1*, which has been shown to regulate milk production phenotypes (Littlejohn *et al*. 2016). RNA was extracted from the four pbMEC cell lines grown on plastic at passages 1, 2, and 3 and qPCR was conducted on these samples. All genes were expressed during at least one of the passages, with expression levels in passage 1 generally the highest (Fig. 4). The expression levels of all the target genes gradually decreased in subsequent passages. Of particular note, *CSN2* and *PAEP* expression was at very low levels after P1. This observation aligns with previously characterised epithelial lines, reporting reduced milk-related gene expression after a few passages. The statistical significance of the differences in the relative expression between passages is summarised in Supplementary Table 1 of the supplementary materials.

### Transfection and CRISPR-Cas9 editing of pbMECs

**Figure 5.**
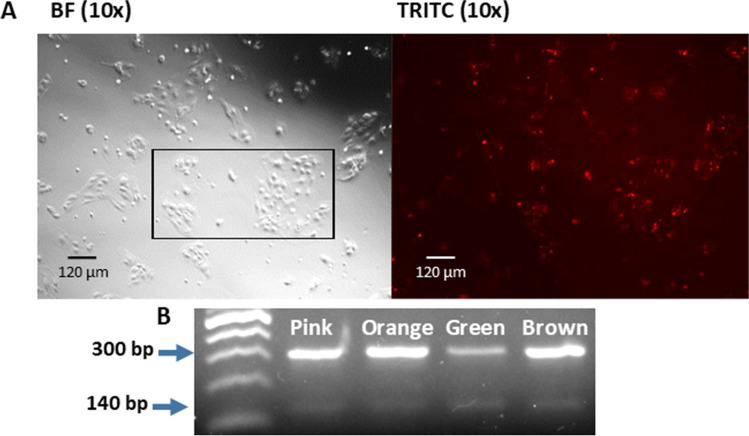
**Efficient transfection and CRISPR-Cas9 editing of pbMECs.** Transfection and editing of pbMEC cell lines with a dual guide simultaneous cut strategy targeting *DGAT1*. (*A*) Transfection efficiency of the Brown line was assessed by transfection of ATTO-labelled tracrRNA into pbMECs using CRISPRMAX lipofection. Bright-field and fluorescent photos were taken of the same frame for each transfection. The *black squared outline* indicates the region of cells counted to determine transfection efficiency. (*B*) Four pbMEC lines were transfected with an RNP complex containing dual guide RNAs targeting *DGAT1,* which were designed to delete 181 nucleotides from the gene. PCR products run on a 1.5% agarose gel demonstrate the expected genome deletion product as a relatively faint band at 138 bp, in addition to the amplicon at 318 bp derived from the cells without the deletion. Gel has been cropped for conciseness; full gel is found in Supplementary Fig. S1. In the *lanes* from left to right, cell lines were *Pink*, *Orange*, *Green*, and *Brown.*

To assess the CRISPR-Cas9 editing capability of the pbMECS, we trialled and optimised several transfection methods. First, we tested the efficiency of the transfection reagent CRISPRMAX to transfect cells with ATTO-labelled tracrRNA. Cells were visualised 24 h post CRISPRMAX-mediated transfection of ATTO-labelled tracrRNA. All lines had a high level of transfection, demonstrated by the presence of ATTO dye within individual cells in the Brown line (Fig. 5*A*). The cells within the outlined area of Fig. 5*A* were counted, and the number of red cells were counted in the same region in the TRITC-filtered image. This gave an estimated transfection efficiency of 41.89%, with 31 of 74 cells appearing to contain ATTO dye. Given the successful transfection of pbMECs with ATTO-labelled tracrRNA using the CRISPRMAX lipid, it was decided to proceed with direct gene editing. A dual guide simultaneous cut strategy targeting *DGAT1* exon 1 was used with the aim of introducing an exonic deletion. Ribonuclear protein (RNP) complexes were formed with two gRNAs, one targeting cleavage before the Methionine start codon and another targeting a region downstream near the 3′ boundary of exon 1 (Fig. 8*A*). The four pbMEC lines were transfected with the RNP complexes and cells were cultured for a further 48 h. Cells were then harvested, genomic DNA extracted, and a PCR with primers flanking the targeted region was undertaken. Each pool of cells demonstrated a low but equal level of editing in all cell lines, apparent in the relative intensity of the intact and deletion amplicons (Fig. 5*B*).

### pbMEC growth on Matrigel

**Figure 6.**
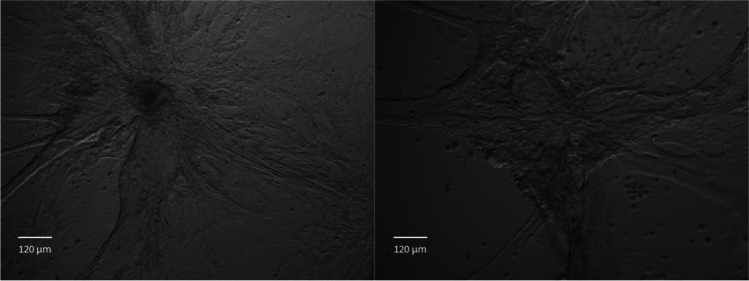
**Light microscope images of pbMECs grown on Matrigel**. Brown pbMECs were grown on Matrigel in differentiation media for 2 wk. Structures were visualised at 10 × magnification using a Nikon Ti-inverted microscope.

Previous studies have shown that established MEC lines maintain lactogenic gene expression when grown on a Matrigel matrix (Prpar Mihevc *et al*. 2014). The Brown line, cultured in differentiation media, was passaged twice on plastic before being plated onto Matrigel. Cells grown on Matrigel had begun to form 3D structures after 4 d of growth, and after 14 d of culturing, duct-like protrusions connected several mammosphere-like structures in the wells (Fig. 6).

**Figure 7.**
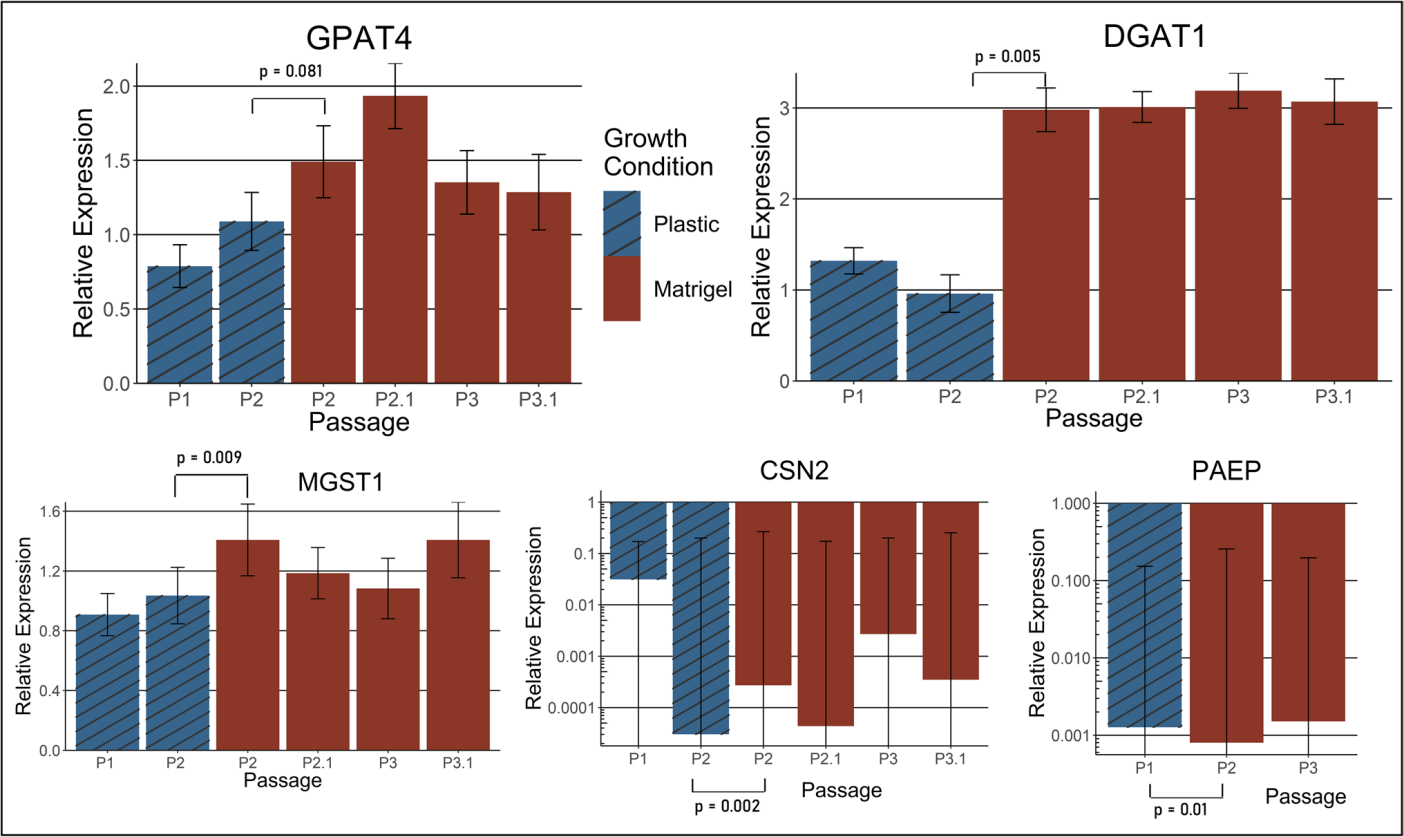
**Lactogenic gene expression of pbMECs is preserved when grown on Matrigel.** Relative expression of five lactation-associated genes in *Green line* pbMECs across passages, grown on different materials. *Blue bars* show the results of cells grown on plastic; *red bars* show the results of cells grown on Matrigel. Results for *CSN2* and *PAEP* are shown on a log scale to improve their resolution. Gene expression is relative to freshly thawed, uncultured biopsy cells, which always having a relative expression of 1, are not plotted. A one-tail two-sample equal variance *t*-test was used to calculate the *p*-values shown. *Error bars* show one standard deviation.

To assess the relative expression over time of the five genes of interest by qPCR, RNA was harvested from freshly thawed Green pbMECs and cells at passages 1 and 2, grown on plastic and Matrigel (Fig. 1). The gene expression results from the freshly thawed, uncultured biopsy cells were used as the control to which the other passages’ gene expression was normalised. As the control always has a relative expression of 1, this result is not plotted on the graphs. The expression levels of the selected genes at passages 1 and 2 cultured on plastic are concordant with the results presented in Fig. 4. Here we found that cellular expression of *CSN2* and *PAEP* decreased across successive passages when the cells were cultured on plastic, while for *GPAT4*, *DGAT1*, and *MGST1,* expression remained stable (Fig. 7). The relative expression levels of all the selected genes for cells grown on Matrigel at passage 2 were higher than cells grown on plastic at passage 1 or 2 (Fig. 7). This difference in gene expression between culturing conditions was significant for *DGAT1*, *MGST1*, *and CSN2*. With the exception of *PAEP* expression, passaging of the 3D structures between wells containing Matrigel (P2.1 and P3.1) either resulted in increased or stable transcript levels compared to the previous passage. Relative expression of *GPAT4*, *DGAT1*, and *MGST1* for cells grown and passaged on Matrigel was higher than for P1 cells grown on plastic. Relative β-casein expression increased at passages 2 and 3 with cells cultured on Matrigel relative to plastic in subsequent passages, but the expression was not restored to the level seen at P1.

### Knock-out of *DGAT1* in pbMECs results in a decrease in gene expression and a change in phenotype

CRISPR-Cas9 editing efficiency of pbMECs using a dual guide RNA approach was relatively low, as determined in a semi-quantitative way by the visualised intensity of the PCR product observed on the agarose gel, representing amplification of the deletion template (Fig. 5*B*). Therefore, to increase the proportion of edited cells in a population, ATTO-labelled tracrRNA was included in the RNP transfection and the cells were sorted using fluorescence-activated cell sorting (FACS). The cells were detached from the plate 24 h after transfection, counterstained with DAPI (to indicate cell viability) and separated by their ATTO positive (41,096 cells) or ATTO negative (336,400 cells) status by an Aria FACS machine. These results indicate that 12.2% of the cells were successfully transfected with enough ATTO-labelled tracrRNA to meet the FACS sorting threshold level.

The ATTO positive population of cells that had been edited and sorted, the RNP only edited and unsorted cells, and unedited control cells were grown for approximately three cell doublings before being plated onto Matrigel-coated wells and cultured for 2 wk. To determine if FACS increased the proportion of edited cells, PCR using primers flanking the expected CRISPR-Cas9-mediated deletion in *DGAT1* was performed on each cell population.

**Figure 8.**
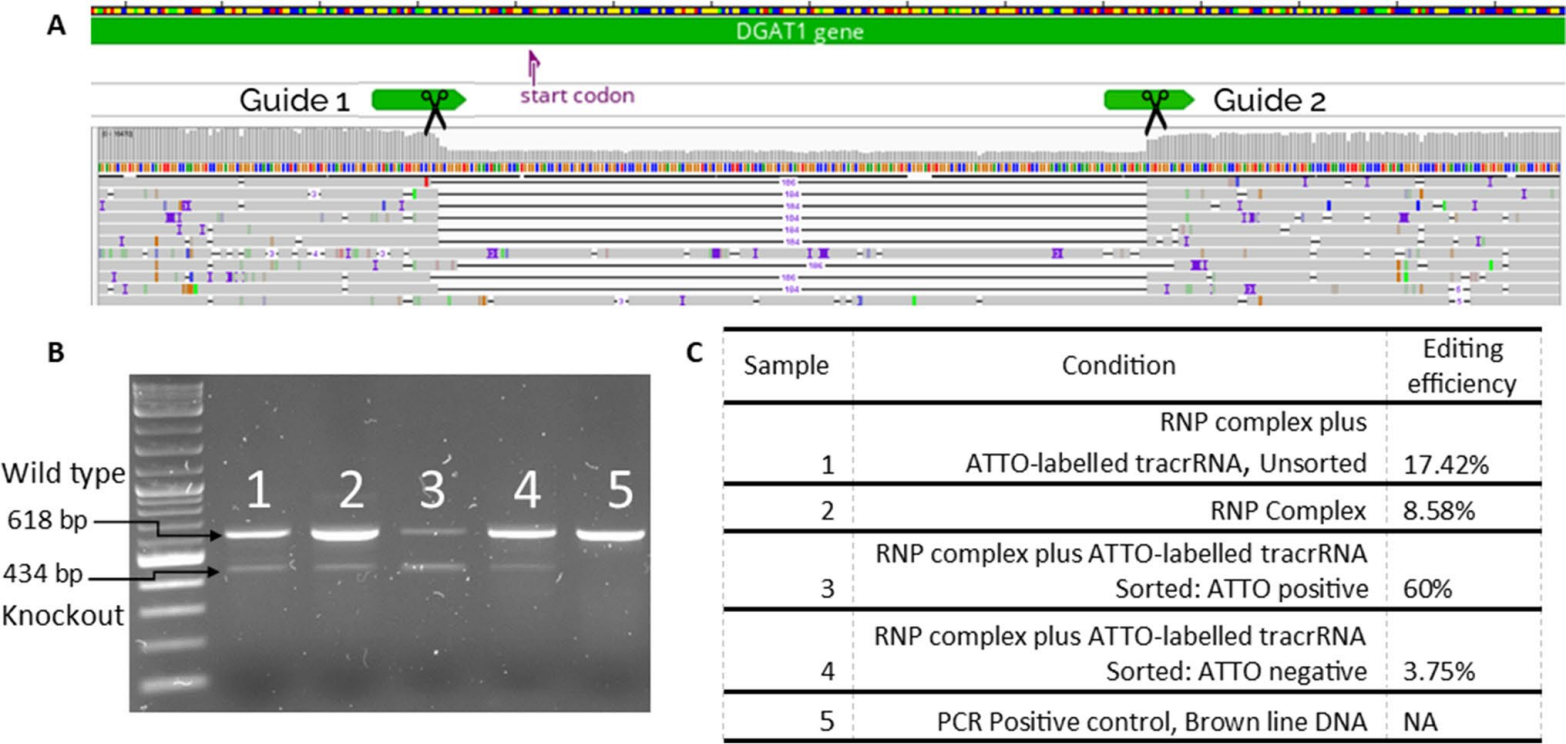
**Sequencing of the PCR amplicon from pbMECs edited **with the *DGAT1* dual guide knock-out establishes editing efficiency. Results from the DGAT1 knock-out experiment on *Brown line* pbMECs. (*A*) Guide placement on the *DGAT1* gene (Geneious) with aligned minION sequencing reads below (IGV) showing reduced read depth over the knock-out region in the ATTO positive group of cells. The reads below demonstrate evidence of the expected 164 base pair deletion. (B) PCR products with primers flanking the DGAT1 knock-out region from each cell population. Gel has been cropped for conciseness; full gel is found in Supplementary Fig. S2. *Lane 1*: 1 kb Plus Ladder (NEB). *Lane 2*: PCR products of DNA from cells transfected with RNP complex and ATTO-labelled tracrRNA, original unsorted population. *Lane 3*: PCR products of DNA from cells transfected with only the RNP complex (no ATTO label), unsorted. *Lane 4*: PCR products of DNA from cells transfected with RNP complex and ATTO-labelled tracrRNA that contained ATTO when sorted. *Lane 5*: PCR products of DNA from cells transfected with RNP complex and ATTO-labelled tracrRNA that did not contain ATTO when sorted. *Lane 6*: PCR products of DNA from wild-type Brown pbMECs. (*C*) *Table* shows the estimated editing efficiency in each cell population as determined by aligned sequence depth of minION sequencing of the PCR amplicons.

The full-length PCR product was separated by gel electrophoresis from the shorter fragments, which indicate dual guide mediated deletion. Figure 8*B* shows a more prominent PCR band corresponding to the deletion in the ATTO positive, edited and sorted cell population (Fig. 8*B*, lane 4), compared with the knock-out band seen in the original unsorted, edited cell population (Fig. 8*B*, lane 2).

**Figure 9.**
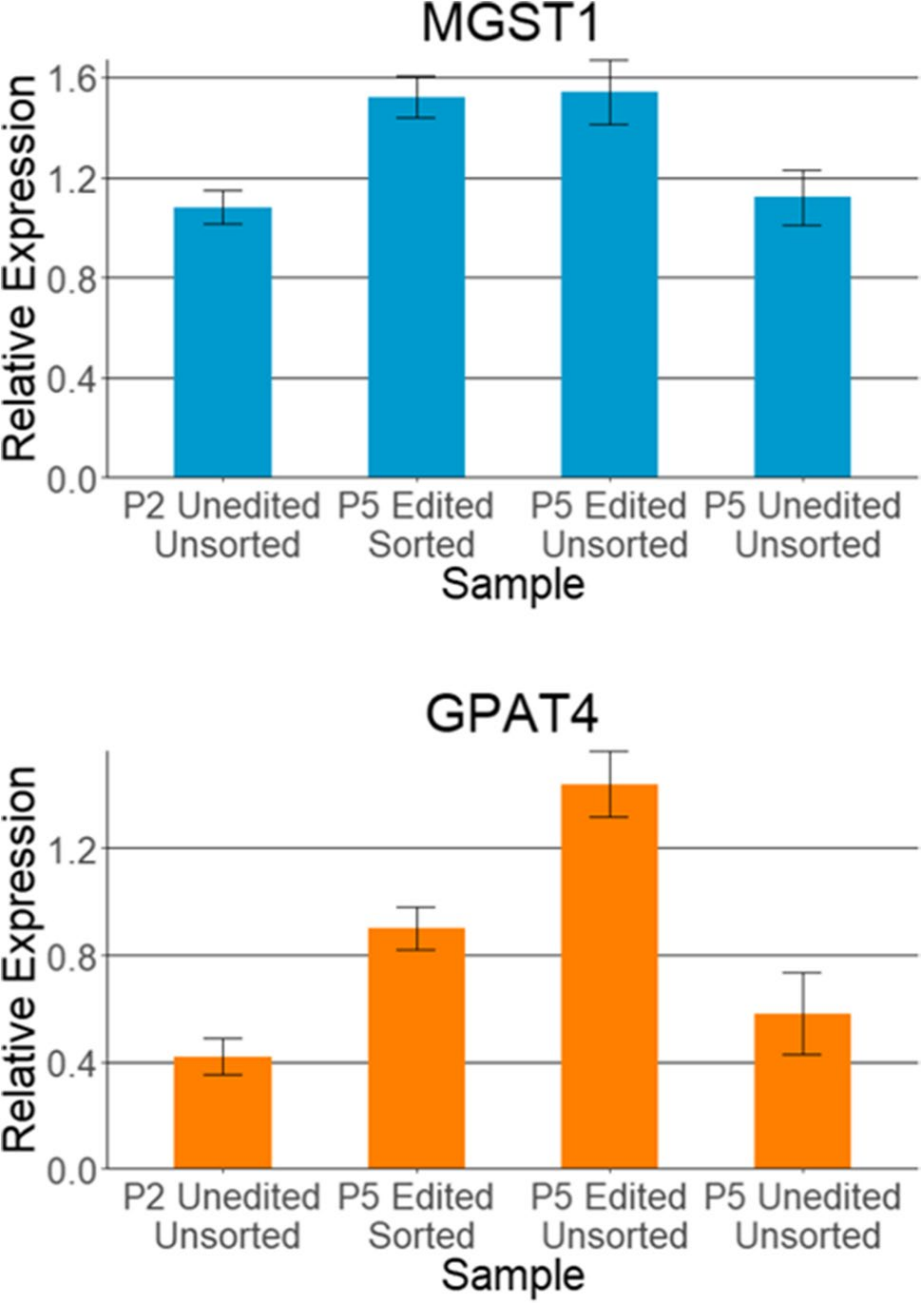
Expression of *MGST1* and *GPAT4* in pbMECs edited with DGAT1 knock-out, sorted via FACS and grown on plastic. Relative expression of *MGST1* and *GPAT4* genes in Brown pbMECs before and after transfection and sorting. The passage 2 cells were wild type unsorted cells, and the passage 5 cells were either edited and sorted, only edited, or wild type unsorted cells. All cells were grown on plastic. The 125 × dilution result from the standard curve was used as the control. *Error bars* show one standard deviation.

To quantify the proportion of edited alleles in each population, the PCR amplicons were sequenced via minION (Fig. 8*C*). The editing frequency was calculated by averaging the read depth across the deletion site compared to the unedited genomic context. minION sequencing of DGAT1 PCR amplicons can be found here: PRJNA862600. Sequencing of the amplicons derived from the ATTO-labelled tracrRNA unsorted cells (Fig. 8*B*, lane 2) demonstrated a 17.42% editing efficiency. The sequencing from the same cells that were ATTO positive after sorting using FACS (Fig. 8*B*, lane 4) showed 60% editing. As expected, the negatively sorted cells from the same pool (ATTO negative), had a much lower editing frequency of 3.75% (Fig. 8*B*, lane 4) compared to the pre-sorted pool of cells. The editing frequency of the unsorted cells containing only the RNP complex (No ATTO label) was 8.58%. Sorting the cells containing ATTO-Labelled tracrRNA improved the proportion of edited cells in the population by 42.58%. A preliminary experiment assessed the maintenance of the expression of lactogenic genes *GPAT4* and *MGST1* after editing and sorting had occurred, and cells were grown on plastic. Figure 9 demonstrates that after the primary cells had undergone editing and sorting, they maintained expression of both *GPAT4* and *MGST1* genes to the same level or higher than the cells which had not undergone these methods. These results suggest that the primary cells can maintain their lactogenic gene expression profile even after the dual disturbance of lipofection and sorting.

**Figure 10.**
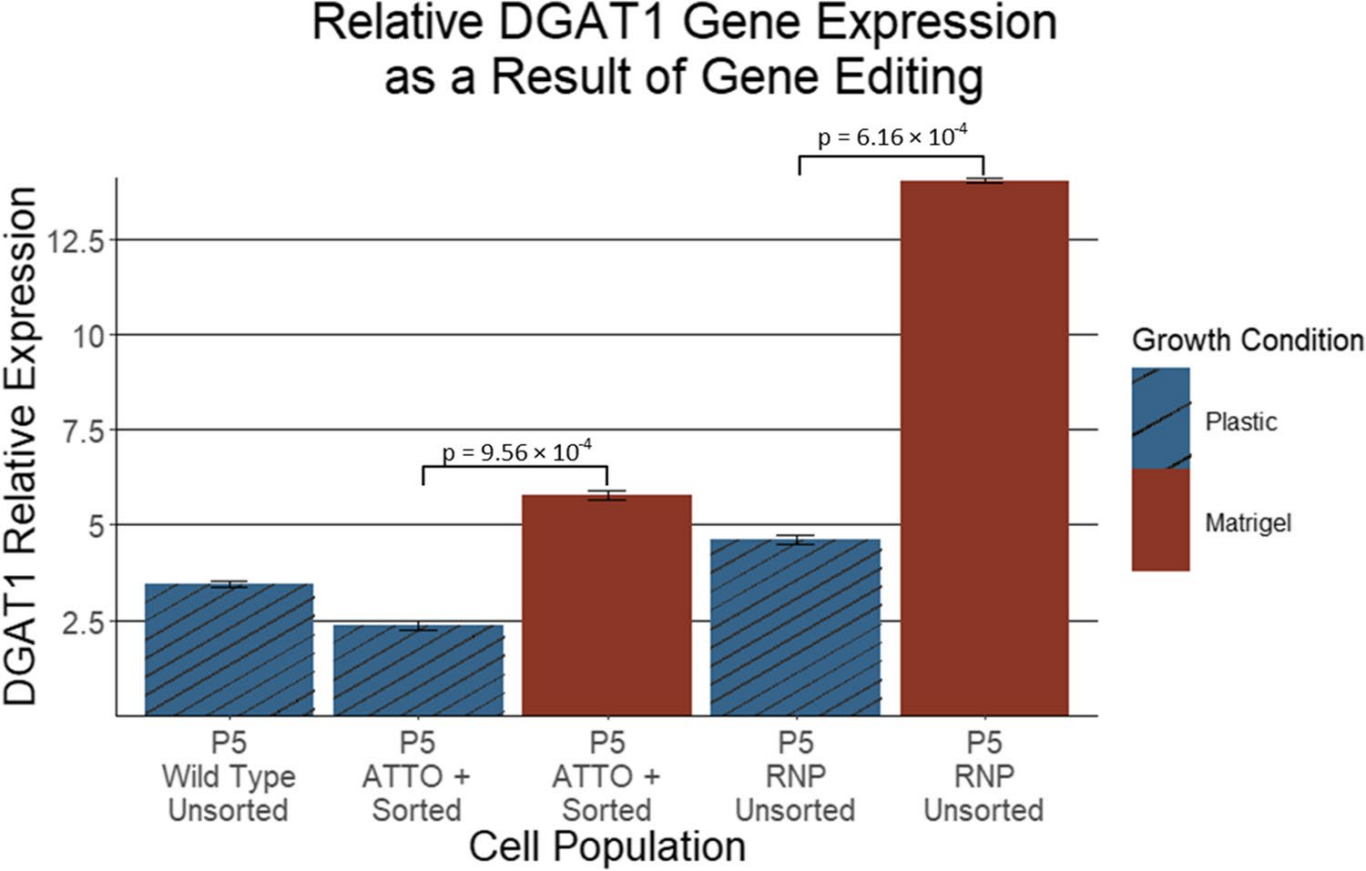
DGAT1 expression of pbMECs edited with *DGAT1* dual guide knock-out, FACS sorted and grown on Matrigel and plastic. Relative gene expression of *DGAT1* in edited and non-edited pbMECs at passage 5. ATTO + refers to the cells that were ATTO positive during FACS sorting and RNP refers to cells that were transfected with the RNP complex and not sorted. The 125 × dilution result from the standard curve was used as the control. *Blue bars* indicate *DGAT1* expression levels of cells grown on plastic and *red bars* indicate *DGAT1* expression levels of cells grown on Matrigel. A one-tail, two-sample equal variance *t*-test was used to calculate the *p*-values shown. *Error bars* show one standard deviation.

To determine if the deletion of the targeted region of *DGAT1* exon 1 influenced the abundance of functional *DGAT1* mRNA, qPCR was conducted on RNA extracted from the sorted and unsorted cells at passage 5 grown on Matrigel and on plastic (Fig. 10). When grown on plastic, *DGAT1* expression was reduced in cells that had undergone DGAT1 knock-out editing and ATTO positive sorting (ATTO + Sorted on plastic; relative expression: 2.36 ± 0.1) compared to the unsorted DGAT1 knock-out edited cells (RNP only on plastic; relative expression: 4.63 ± 0.12) and wild type cells (Wild-type unsorted on plastic; relative expression: 3.45 ± 0.08). Similarly, *DGAT1* expression was reduced in edited and sorted ATTO-positive cells grown on Matrigel (ATTO + Sorted on Matrigel; relative expression: 5.8 ± 0.11) compared with edited but unsorted cells grown on Matrigel (RNP only on Matrigel; relative expression: 14.04 ± 0.06). Potential off-target regions in the ATTO plus RNP transfected cells (well A1) were analysed by PCR amplification and Sanger sequencing. Supplementary Figure S3 shows the Sanger traces of the potential guide binding regions. There was no off-target editing found at any of the top four ranked potential off-target sequences. We see the sequence traces of the edited cells are an exact match to the wild-type sequence, across the off-target regions, indicating no cutting at these locations.

## Discussion

The culturing of primary cells in vitro is a technique that facilitates the observation of transcriptomic and phenotypic changes in cells in response to many different experimental perturbations — including gene editing (Ge *et al*. 2021). Cell models with physiological features closely representing the tissue in question are more desirable for this purpose. Investigating the influence of genetic variants or the introduction of transgenes on milk production traits is an essential tool allowing, for example, the identification of markers for productivity selection (Lopdell *et al*. 2017; Pegolo *et al*. 2018). Targeted genetic changes introduced by CRISPR-Cas9 editing in primary cell models may help to elucidate the effects of these variants on phenotypes of interest including fat and protein percentage and overall milk yield. The major limitation of cell culture models of lactating mammary tissue is the loss of expression of lactation-related genes (especially those that encode milk proteins) after only a few passages (Jedrzejczak and Szatkowska 2014). This characteristic puts a time constraint on experiments and limits investigation to early cell passages. It may also decrease the reproducibility of variant effects between primary lines that do not have stable gene expression.

Characterisation by ICC of the four primary cell lines derived from tissue biopsies revealed they were all predominantly epithelial in nature (Fig. 3). Additionally, there was no evidence of fibroblasts outcompeting epithelial cells during our experiments, which may happen as the extraction process does not remove all fibroblasts. All four primary pbMEC cell lines were successfully transfected using CRISPRMAX lipofectamine reagent, and delivery of guide RNAs in a ribonuclear complex resulted in targeted gene editing. The gene expression profile of the key milk-related genes grown on plastic over three passages was found to be similar to previously characterised pbMEC lines, where there is a rapid loss of expression of milk proteins. In particular, casein and beta-lactoglobulin expression decreased substantially after only a few passages (Fig. 4). As others have reported, we found that when cultured on Matrigel with prolactin, milk protein gene expression is maintained or re-established, at least to some extent, for four passages and multiple weeks of culturing (Sun *et al*. 2005; Riley *et al*. 2010). In addition, cells grown on Matrigel formed 3D structures visible under the light microscope, as previously reported (Rose *et al*. 2002; Anand 2012).

We have demonstrated the use of FACS to enrich for a population of gene-edited primary cells based on their ability to uptake CRISPR reagents. This has wide-ranging applications when considering gene editing. The methods described here provide a system of protocols for maintaining the expression of milk protein and fat synthesis genes for multiple weeks, in addition to enhancing the proportion of edited cells in a population. When combined, these methods may give greater certainty that changes in gene expression after gene editing will be observable and more physiologically relevant compared to primary cells grown on plastic or immortalised cell lines. This technique may be applied to many different cell types to investigate any type of genetic perturbation.

We chose *DGAT1* as a functional knock-out gene target for the development of the methodology as it is a critical enzyme for triglyceride synthesis, and variants within this gene are known to significantly influence fat yield in milk (Fink *et al*. 2020). Specifically, *DGAT1* is responsible for the addition of the third fatty acid chain converting diacylglycerol to triacylglycerol. *DGAT1* expression was relatively high and consistent in cells grown on Matrigel, allowing resolution of the changes in gene expression after deletion editing. Post-editing FACS improved the proportion of pbMEC cells with the knock-out from 17.4 to 60%. The sorted edited cells had 41% lower *DGAT1* relative expression than the unsorted cells. Edited cells grown on Matrigel showed a significantly higher level of *DGAT1* expression compared to their equivalent population growing on plastic. We also demonstrated that the cells could go through at least four cell doublings and still recover their *DGAT1* gene expression. Growth on Matrigel also allowed initial gene expression levels to be maintained for longer, which dramatically expanded the time frame during which we could assess the effects of gene editing on edited primary cell cultures.

Other attempts to create a CRISPR-Cas9 knock-out in lactating mammary epithelial lines have yielded lower editing efficiencies than the presented results. Zhang *et al*. 2021, overcame this by isolating a single Goat mammary epithelial cell (GMEC) TPH1 knock-out clone that was confirmed to have a knock-out in both alleles before further experiments were conducted to assess intracellular calcium levels (Zhang *et al*. 2021). These cells would have gone through tens of doublings before reaching sufficient numbers to be used in experiments which may limit the investigation of milk protein gene expression. Other authors have used a similar approach to assess the effect of microRNA and gene knock-outs on the expression of fatty acid metabolism-related genes (Tian *et al*. 2018; Huang *et al*. 2020). They observed changes in the expression of fatty acid synthesis genes, including *DGAT1*, in their primary GMEC clones with the knock-out compared to a WT clone. It is unclear how physiologically relevant these results are, given the high number of cell doubling these cell populations have gone through and the culturing of the cells on plastic.

The efficiency of the introduction of specific mutations by homology-directed repair (HDR) is likely to be lower than the efficiency of introducing knock-out deletions (Prill and Dawson 2020). Editing rates are affected by the delivery method, the target gene sequence, and the HDR template used (Prill and Dawson 2020). However, methods such as the FACS sorting applied here should have relevance to HDR-based methods and result in editing-enriched populations.

In summary, the methods and results presented here demonstrate the potential for creating a CRISPR-Cas9-edited primary cell line with a high proportion of edited cells that can maintain initial levels of lactogenic gene expression. The combination of FACS and culturing of cells on Matrigel enables the investigation of the impacts of variants introduced via gene editing on the expression of central lactogenic genes in primary cells for extended periods.

## Supplementary Information

Below is the link to the electronic supplementary material.

Supplementary file1 (JPG 44 KB)

Supplementary file2 (JPG 45 KB)

Supplementary file3 (DOCX 126 KB)

Supplementary file4 (DOCX 20 KB)

## Data Availability

Raw qPCR data and calculations can be found at https://doi.org/10.7910/DVN/YATECZ. minION sequencing of DGAT1 PCR amplicons can be found here: PRJNA862600.

## Abbreviations

*pbMEC*: Primary bovine mammary epithelial cell
*CRISPR*: Clustered regularly interspaced short palindromic repeats
*crRNA*: CRISPR ribonucleic acid
*DGAT1*: Diacylglycerol O-acyltransferase 1
*gRNA*: Guide ribonucleic acid
*MAC-T*: Primary bovine mammary alveolar cells immortalised by stable transfection with SV-40 large T-antigen
*SpCas9*: (CRISPR)-associated S. pyogenes nuclease Cas9
*tracrRNA*: Trans-activating CRISPR ribonucleic acid
*FACS*: Fluorescence-assisted cell sorting
*NHEJ*: Non-homologous end joining
*DMEM*: Dulbecco’s Modified Eagle Medium
*DM*: Differentiation media
*ICC*: Immunocytochemistry
*RNP*: Ribonuclear protein
*HDR*: Homology-directed repair

## References

[CR1] Anand V *et al* (2012) Establishment and characterization of a buffalo (bubalus bubalis) mammary epithelial cell line. PLoS One 7(7):e40469. 10.1371/journal.pone.0040469 10.1371/journal.pone.0040469 PMC3392245 22792341

[CR2] Boutinaud M, Herve L, Lollivier V (2015). Mammary epithelial cells isolated from milk are a valuable, non-invasive source of mammary transcripts. Front Genet.

[CR3] Concordet JP, Haeussler M (2018). CRISPOR: intuitive guide selection for CRISPR/Cas9 genome editing experiments and screens. Nucleic Acids Res.

[CR4] Ebrahimi V, Hashemi A (2020). Challenges of in vitro genome editing with CRISPR/Cas9 and possible solutions: a review. Gene.

[CR5] Edick AM, Audette J, Burgos SA (2021). CRISPR-Cas9-mediated knockout of GCN2 reveals a critical role in sensing amino acid deprivation in bovine mammary epithelial cells. J Dairy Sci.

[CR6] Fink T (2020). A new mechanism for a familiar mutation - bovine DGAT1 K232A modulates gene expression through multi-junction exon splice enhancement. BMC Genomics.

[CR7] Fu YW (2021). Dynamics and competition of CRISPR-Cas9 ribonucleoproteins and AAV donor-mediated NHEJ, MMEJ and HDR editing. Nucleic Acids Res.

[CR8] Ge L (2021). Myostatin site-directed mutation and simultaneous PPARγ site-directed knockin in bovine genome. J Cell Physiol.

[CR9] Hernandez LL (2008). Evaluation of serotonin as a feedback inhibitor of lactation in the bovine. J Dairy Sci.

[CR10] Hillreiner M (2017). Establishment of a 3D cell culture model of primary bovine mammary epithelial cells extracted from fresh milk. Vitro Cell Dev Biol Anim.

[CR11] Huang L, Tian H, Luo J, Song N, Wu J (2020). CRISPR/Cas9 based knockout of miR-145 affects intracellular fatty acid metabolism by targeting INSIG1 in goat mammary epithelial cells. J Agric Food Chem.

[CR12] Huynh HT, Robitaille G, Turner JD (1991). Establishment of bovine mammary epithelial cells (MAC-T): an in vitro model for bovine lactation. Exp Cell Res.

[CR13] Jedrzejczak M, Szatkowska I (2014). Bovine mammary epithelial cell cultures for the study of mammary gland functions. Vitro Cell Dev Biol Anim.

[CR14] Kibbey MC (1994). Maintenance of the EHS sarcoma and Matrigel preparation. J Tissue Cult Methods.

[CR15] Kumura H (2001). Primary culture of porcine mammary epithelial cells as a model system for evaluation of milk protein expression. Biosci Biotechnol Biochem.

[CR16] Littlejohn MD (2016). Sequence-based association analysis reveals an MGST1 eQTL with pleiotropic effects on bovine milk composition. Sci Rep.

[CR17] Liu G, Zhang Y, Zhang T (2019). Computational approaches for effective CRISPR guide RNA design and evaluation. Comput Struct Biotechnol J.

[CR18] Liu M (2019). Methodologies for improving HDR efficiency. Front Genet.

[CR19] Lopdell TJ (2017). DNA and RNA-sequence based GWAS highlights membrane-transport genes as key modulators of milk lactose content. BMC Genomics.

[CR20] Mroue R, Bissell MJ (2013). Three-dimensional cultures of mouse mammary epithelial cells. Methods Mol Biol.

[CR21] Pal K, Grover PL (1983). A simple method for the removal of contaminating fibroblasts from cultures of rat mammary epithelial cells. Cell Biol Int Rep.

[CR22] Pegolo S (2018). Integration of GWAS, pathway and network analyses reveals novel mechanistic insights into the synthesis of milk proteins in dairy cows. Sci Rep.

[CR23] Prill K, Dawson JF (2020). Homology-directed repair in zebrafish: witchcraft and wizardry?. Front Mol Biosci.

[CR24] Prpar Mihevc S, Ogorevc J, Dovc P (2014). Lineage-specific markers of goat mammary cells in primary culture. Vitro Cell Dev Biol Anim.

[CR25] Riley LG (2010). The influence of extracellular matrix and prolactin on global gene expression profiles of primary bovine mammary epithelial cells in vitro. Anim Genet.

[CR26] Rose MT (2002). In vitro differentiation of a cloned bovine mammary epithelial cell. J Dairy Res.

[CR27] Sakamoto K (2005). Growth hormone acts on the synthesis and secretion of α-casein in bovine mammary epithelial cells. J Dairy Res.

[CR28] Sun YL, Lin CS, Chou YC (2005). Gene transfection and expression in a primary culture of mammary epithelial cells isolated from lactating sows. Cell Biol Int.

[CR29] Tian H (2018). CRISPR/Cas9-mediated stearoyl-CoA desaturase 1 (SCD1) deficiency affects fatty acid metabolism in goat mammary epithelial cells. J Agric Food Chem.

[CR30] Walker CG, Meier S, Mitchell MD, Roche JR, Littlejohn M (2009). Evaluation of real-time PCR endogenous control genes for analysis of gene expression in bovine endometrium. BMC Mol Biol.

[CR31] Xu K, Buchsbaum RJ (2012) Isolation of mammary epithelial cells from three-dimensional mixed-cell spheroid co-culture. J Vis Exp (62):3760. 10.3791/3760 10.3791/3760 PMC3466662 22566026

[CR32] Zavizion B, van Duffelen M, Schaeffer W, Politis I (1996). Establishment and characterization of a bovine mammary epithelial cell line with unique properties. Vitro Cell Dev Biol Anim.

[CR33] Zhang Z (2021). CRISPR/Cas9-mediated tryptophan hydroxylase 1 knockout decreases calcium transportation in goat mammary epithelial cells. Biochem Eng J.

[CR34] Zhao K, Liu H, Zhou M, Liu J (2010). Establishment and characterization of a lactating bovine mammary epithelial cell model for the study of milk synthesis. Cell Biol Int.

[CR35] Zhou Y, Akers RM, Jiang H (2008). Growth hormone can induce expression of four major milk protein genes in transfected MAC-T cells. J Dairy Sci.

