# Supplementary figures and images for: Successful editing and maintenance of lactogenic gene expression in primary bovine mammary epithelial cells
Source: In Vitro Cell Dev Biol Anim. 2023 Jun 6;59(5):316–30. doi: 10.1007/s11626-023-00762-6 (PMC10322751; doi:10.1007/s11626-023-00762-6)

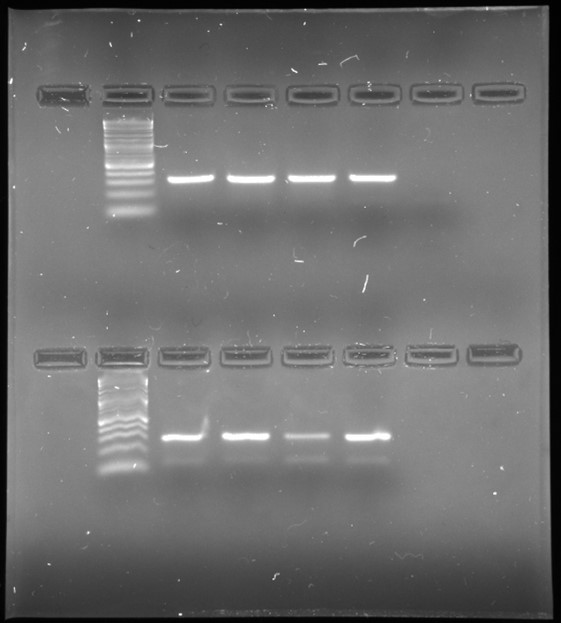

Supplement: Supplementary file 1 — Supplementary file1 (JPG 44 KB) [file 11626_2023_762_MOESM1_ESM.jpg]

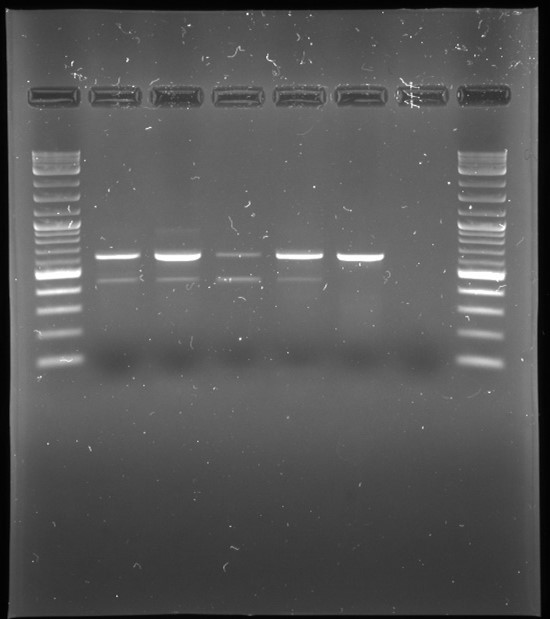

Supplement: Supplementary file 2 — Supplementary file2 (JPG 45 KB) [file 11626_2023_762_MOESM2_ESM.jpg]
