# Supplementary material for: Successful editing and maintenance of lactogenic gene expression in primary bovine mammary epithelial cells
Source: In Vitro Cell Dev Biol Anim. 2023 Jun 6;59(5):316–30. doi: 10.1007/s11626-023-00762-6 (PMC10322751; doi:10.1007/s11626-023-00762-6)
Supplement: Supplementary file 3 — Supplementary file3 (DOCX 126 KB) [file 11626_2023_762_MOESM3_ESM.docx]

|  | Guide 1 | Guide 2 |
| --- | --- | --- |
| Consensus  Wild Type  Guide  Sanger | 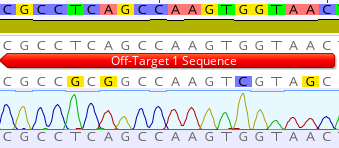 | 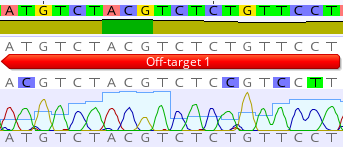 |
| Consensus  Wild Type  Guide  Sanger | 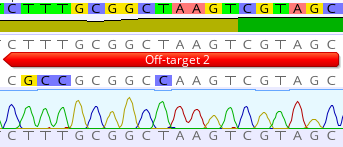 | 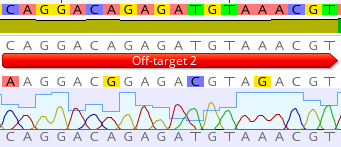 |
| Consensus  Wild Type  Guide  Sanger | 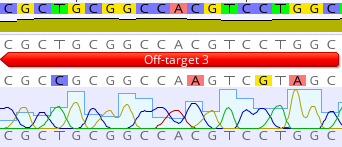 | 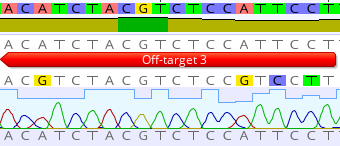 |
| Consensus  Wild Type  Guide  Sanger | 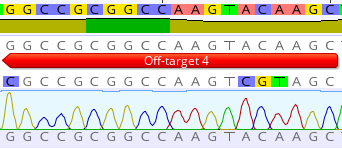 | 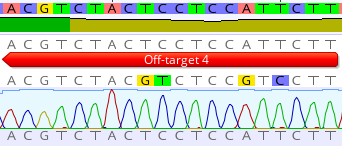 |

**Supplementary Figure S3: Sanger sequencing traces of the potential off-target guide binding regions for two DGAT1 knockout guides.** The top four potential off-target regions for each guide were sequenced for each guide. These regions were amplified using PCR and the amplicons were Sanger sequenced. The sequences shown in each panel from top to bottom are: Consensus sequence, wild type sequence, the original guide sequence with deviations highlighted and sanger sequence of those regions.
