# Supplementary material for: Successful editing and maintenance of lactogenic gene expression in primary bovine mammary epithelial cells
Source: In Vitro Cell Dev Biol Anim. 2023 Jun 6;59(5):316–30. doi: 10.1007/s11626-023-00762-6 (PMC10322751; doi:10.1007/s11626-023-00762-6)
Supplement: Supplementary file 4 — Supplementary file4 (DOCX 20 KB) [file 11626_2023_762_MOESM4_ESM.docx]

**Supplementary Table 1:** **Statistical comparison of the relative expression** between the different passages of each cells line, for five key lactational genes. A one-tail two-sample equal variance t-test was used to calculate the *P*-values shown.

|  |  |  | **P-Values** |  |  |
| --- | --- | --- | --- | --- | --- |
| Sample | **GPAT4** | **CSN2** | **DGAT1** | **PAEP** | **MGST1** |
| Pink P1-P2 | 0.073 | 1.36E-05 | 0.129 | 0.004 | 0.098 |
| Pink P2-P3 | 0.028 | 1.55E-03 | 0.014 | 0.006 | 5.77E-05 |
| Pink P1 - P3 | 0.052 | 1.21E-05 | 0.212 | 4.74E-04 | 2.76E-03 |
| Orange P1 -P2 | 0.034 | 2.05E-06 | 0.248 | 1.85E-04 | 0.302 |
| Orange P2 -P3 | 0.070 | 3.84E-03 | 0.001 | 0.003 | 2.86E-04 |
| Orange P1 -P3 | 0.024 | 5.35E-06 | 0.001 | 4.99E-05 | 4.01E-04 |
| Green P1 - P2 | 0.001 | 9.08E-06 | 0.136 | 2.39E-03 | 0.005 |
| Green P2 - P3 | 0.151 | 1.40E-03 | 0.076 | 0.048 | 6.49E-05 |
| Green P1 - P3 | 0.015 | 9.26E-06 | 0.013 | 2.52E-05 | 7.61E-06 |
| Brown P1 - P2 | 0.195 | 1.28E-05 | 0.257 | 3.61E-05 | 0.003 |
| Brown P2 - P3 | 0.117 | 1.99E-05 | 0.008 | 0.011 | 1.52E-04 |
| Brown P1- P3 | 0.018 | 4.17E-07 | 0.003 | 1.09E-03 | 1.12E-04 |

**Supplementary Table 2: Potential off target regions for the DGAT1 knockout guides.** This table contains the potential off-target regions ranked by cutting frequency determination (CFD) score, the sequence of the potential binding site, where the off-target region is found in the genome and the forward and reverse primers used to complete PCR amplification of the region surrounding the off-target site.

| Off target # | Sequence | Gene | CFD Score | Forward primer | Reverse Primer |
| --- | --- | --- | --- | --- | --- |
| Guide 1 |  |  |  |  |  |
| 1 | GTTACCACTTGGCTGAGGCG | [intergenic:LRRC23-SPSB2](http://genome.ucsc.edu/cgi-bin/hgTracks?db=bosTau9&position=chr5:103577383-103577405) | 0.454 | GAAAGGGTTAATTCTGAGCTTGGG | CGAGCCGAAAGTTACCGAAG |
| 2 | GCTACGACTTAGCCGCAAAG | [intron:FMN1](http://genome.ucsc.edu/cgi-bin/hgTracks?db=bosTau9&position=chr10:29459706-29459728) | 0.276 | CACACATCTGCTGCTTTATTCC | GGTCTGTTTAGCAGGAAAAGGA |
| 3 | GCCAGGACGTGGCCGCAGCG | [intergenic:TMEM119-ISCU](http://genome.ucsc.edu/cgi-bin/hgTracks?db=bosTau9&position=chr17:64351351-64351373) | 0.148 | AATGGCCGTAAGAGAGAATAGG | CTTTAGCCCCAGAGATACACC |
| 4 | GCTTGTACTTGGCCGCGGCC | [intergenic:TMEM119-ISCU](http://genome.ucsc.edu/cgi-bin/hgTracks?db=bosTau9&position=chr17:64351351-64351373) | 0.109 | TGATGGCATCTCCCCCAATC | TGGAGGACTTCTGGTCAAACAT |
| Guide 2 |  |  |  |  |  |
| 1 | AGGAACAGAGACGTAGACAT | [intergenic:DEDD2-POU2F2](http://genome.ucsc.edu/cgi-bin/hgTracks?db=bosTau9&position=chr18:51105448-51105470) | 0.514 | TTTGCATAGCAGCGTACACA | GGAGACCAAATGAACAGCTGAT |
| 2 | CAGGACAGAGATGTAAACGT | [intergenic:ZP4-5S_rRNA](http://genome.ucsc.edu/cgi-bin/hgTracks?db=bosTau9&position=chr28:10740420-10740442) | 0.461 | ACTGTTCGTAGAGTGAGGAAAC | CACCTATTTGCCTTATGCCATG |
| 3 | AGGAATGGAGACGTAGATGT | [intron:SMAD9](http://genome.ucsc.edu/cgi-bin/hgTracks?db=bosTau9&position=chr12:24763623-24763645) | 0.429 | ACGTGGGTAGTGGGTCTATATT | CTCTAGTGAGGTGGATGAACC |
| 4 | AAGAATGGAGGAGTAGACGT | [intergenic:LARGE1-ISX](http://genome.ucsc.edu/cgi-bin/hgTracks?db=bosTau9&position=chr5:72393316-72393338) | 0.388 | CCAGGAAGAAGCCGAAATGAAA | GACTTTGTTTTCCTGAACCACC |
